# Estimating the prevalence of functional exonic splice regulatory information

**DOI:** 10.1007/s00439-017-1798-3

**Published:** 2017-04-12

**Authors:** Rosina Savisaar, Laurence D. Hurst

**Affiliations:** The Milner Centre for Evolution, Department of Biology and Biochemistry, University of Bath, Bath, BA2 7AY UK

## Abstract

In addition to coding information, human exons contain sequences necessary for correct splicing. These elements are known to be under purifying selection and their disruption can cause disease. However, the density of functional exonic splicing information remains profoundly uncertain. Several groups have experimentally investigated how mutations at different exonic positions affect splicing. They have found splice information to be distributed widely in exons, with one estimate putting the proportion of splicing-relevant nucleotides at >90%. These results suggest that splicing could place a major pressure on exon evolution. However, analyses of sequence conservation have concluded that the need to preserve splice regulatory signals only slightly constrains exon evolution, with a resulting decrease in the average human rate of synonymous evolution of only 1–4%. Why do these two lines of research come to such different conclusions? Among other reasons, we suggest that the methods are measuring different things: one assays the density of sites that affect splicing, the other the density of sites whose effects on splicing are visible to selection. In addition, the experimental methods typically consider short exons, thereby enriching for nucleotides close to the splice junction, such sites being enriched for splice-control elements. By contrast, in part owing to correction for nucleotide composition biases and to the assumption that constraint only operates on exon ends, the conservation-based methods can be overly conservative.

**Electronic supplementary material:**

The online version of this article (doi:10.1007/s00439-017-1798-3) contains supplementary material, which is available to authorized users.

## Introduction

A potentially important insight from the past couple of decades of work on mammalian genomes has been that genetic information is not always stored serially, with different kinds of elements arranged one after the other in neatly separated compartments (e.g. promoter compartments, which contain regulatory signals, followed by genic compartments, which contain coding information, with no overlaps between different open reading frames). Instead, our genomes are fundamentally multi-layered: not only can open reading frames overlap each-other (Lazar et al. 1989; Makalowska et al. 2005; Michel et al. 2012; Miyajima et al. 1989; Sanna et al. 2008; Stallmeyer et al. 1999; Veeramachaneni et al. 2004), they also routinely overlap various kinds of regulatory elements (Itzkovitz et al. 2010; Lin et al. 2011; Shabalina et al. 2013). An example of the latter would be a microRNA binding site embedded inside a coding sequence (CDS) (Fang and Rajewski 2011; Forman et al. 2008; Hausser et al. 2013; Hurst 2006; Lewis et al. 2005; Liu et al. 2015). Such overlaps imply that the evolution of CDSs depends not only on selection pressures related to protein structure and function but also on selection on overlapping regulatory signals.

Exonic splice enhancers (ESEs) are the class of regulatory signals whose impact on CDS evolution has been most thoroughly demonstrated [although other kinds of non-coding information have also been studied (e.g. Agoglia and Fraser 2016; Birnbaum et al. 2014; Cakiroglu et al. 2016; Hurst 2006; Itzkovitz et al. 2010; Lin et al. 2011; Liu et al. 2015; Shabalina et al. 2013; Stergachis et al. 2013; Warnecke et al. 2008a; Xing and He 2015)]. ESEs are short RNA motifs that promote the splicing of the exon in which they are contained. They mostly represent binding sites to RNA-binding proteins (RBPs) that contact the exonic regions of the pre-mRNA (Fu and Ares 2014; Lee and Rio 2015). They have been repeatedly shown to be under purifying selection using both divergence (Cáceres and Hurst 2013; Parmley et al. 2006; Sterne-Weiler et al. 2011) and population genetic data (Cáceres and Hurst 2013; Carlini and Genut 2006; Fairbrother et al. 2004; Majewski and Ott 2002), and their disruption can cause disease (e.g. Collin et al. 2008; Lim et al. 2011; Moseley et al. 2002; Ramser et al. 2005; Sterne-Weiler et al. 2011; see Wen et al. 2016 for a database of disease-associated synonymous mutations in general). The effect of ESEs might even extend to the level of protein structure: evidence suggests that protein regions where the underlying RNA sequence contains splice regulatory information have greater rates of structural disorder (Macossay-Castillo et al. 2014; Pancsa and Tompa 2016; Smithers et al. 2015). In addition, amino acid usage in protein regions encoded for by exon ends, where ESE density is highest, appears to be biased by the underlying ESE presence (Parmley et al. 2007). However, despite ample evidence that ESEs are functional and do indeed play a role in CDS evolution, the scale of the phenomenon remains uncertain. How prevalent is functional exonic splice regulatory information (enhancing or inhibitory)? Is the need to preserve such information a major driver of CDS evolution?

Different scenarios are possible. At one end of the spectrum, functional exonic splice regulatory elements could be rare, occurring at well-defined locations within exons and influencing evolutionary rates only very locally. At the other extreme, our exons could be a tight meshwork of negative and positive control signals, and the need to maintain the correct configuration of these elements would pose a constraint on CDS evolution on par with (or perhaps even greater than) that due to selection pressures related to protein structure and function. Where does reality lie on this spectrum? Note that we will not be distinguishing between elements necessary for correct constitutive splicing (including the splice sites themselves) and information involved in establishing regulated alternative splicing patterns.

We will be considering two different approaches to solving this problem. The first is experimental: the researcher introduces mutations at a large number of sites within a model exon and determines how frequently splicing patterns are disrupted as a result, inferring from this the density of exonic splice regulatory information. The second is based on evolutionary conservation: one uses the frequency and rate of evolution of putative splice regulatory motifs to quantify the extent to which their presence constrains CDS evolution.

Unexpectedly, these two approaches yield strikingly different results. Conservation-based analyses have deemed the evolutionary impact of selection on exonic splice regulatory information to be detectable but weak (Cáceres and Hurst 2013; Parmley et al. 2006; Savisaar and Hurst 2017). This implies either that functional exonic splice regulatory elements are rare or, alternatively, that they are frequent but usually only have slight fitness relevance, with high rates of evolutionary turnover. Experimental assays, on the other hand, have found that mutations at anywhere between a fifth (Kergourlay et al. 2014; Thery et al. 2011; Tournier et al. 2008) to over 90% (Julien et al. 2016) of the exonic sites tested can alter splice form ratios. This would suggest that functional splice regulatory elements are highly common in our exons and might therefore play an important role in directing CDS evolution.

In the pages to follow, we will first provide a brief overview of the two approaches. We will then consider four explanations for why the two types of studies arrive at such different conclusions. Briefly, it appears that the discrepancy is partly due to both methods being associated with their own particular sets of caveats, which might lead estimates to diverge. However, the approaches also ask slightly different questions and so it is expected that they would also provide different answers. We hope that the in-depth consideration of the two types of approaches will be useful in informing and unifying future work.

## Two methods for quantifying the prevalence of functional exonic splice regulatory information

### Splicing reporter assays

The first approach makes use of splicing assays (Cooper 2005; Gaildrat et al. 2010). Within the context of a minigene construct, the researcher introduces a series of individual mutations into a model exon. The minigene is cloned into a plasmid vector, the plasmid is transfected into cells and each mutant version of the exon is monitored for differences (compared to wild-type) in the percentage of transcripts where the exon is spliced in (percentage spliced in or PSI). The latter is usually achieved either through reverse transcription polymerase chain reaction (RT-PCR) and imaging of electrophoresis bands, or through next generation sequencing. The final result of the experiment is an estimate for the density of splice regulatory information in the exon, reported either as the fraction of the variants tested that caused a change in PSI or as the fraction of the sites tested where any variants were found to be splice-altering. Table 1 provides an overview of 11 such studies, which altogether investigate the effects of 586 exonic variants in 9 different genes (Di Giacomo et al. 2013; Gaildrat et al. 2012; Julien et al. 2016; Kergourlay et al. 2014; Mueller et al. 2015; Pagani et al. 2003, 2005; Soukarieh et al. 2016; Tajnik et al. 2016; Thery et al. 2011; Tournier et al. 2008).

**Table 1:** Overview of experimental studies on the prevalence of exonic splice information

References	Exon (size)	Variants tested	Proportion of splice-associated sites	Proportion of splice-disrupting variants	Definition of splice alteration	Examples of diseases associated to gene
Pagani et al. (2003)	*CFTR* exon 9 (183 bp)	Variants previously reported in patients and artificial variants	21/29 (~72.4%) (includes some indels and multiple mutations)	32/47 (~68.1%) (includes some indels and multiple mutations)	Undefined	Cystic fibrosis (Cheng et al. 1990)
*Pagani et al.* (2005)	CFTR *exon 12 (87* *bp)*	*Most possible synonymous single base substitutions between positions 13 and 52 in the exon*	*5/12 (*~*41.7%)*	*6/19 (*~*31.6%)*	*Undefined*	*See above*
Tournier et al. (2008)	*MLH1* and *MSH2* (various exons)	Variants (of unknown significance or deleterious) from Lynch syndrome families	13/67 (~19.4%) (includes short indels)	13/67 (~19.4%) (includes short indels)	Determined using a *t* test (distribution from 3 replicate experiments)	Lynch syndrome (Bonadona et al. 2011; Fishel et al. 1993; Bronner et al. 1994)
Thery et al. (2011)	*BRCA1* and *BRCA2* (various exons)	Variants of unknown significance from families undergoing genetic counselling	6/30 (20.0%)	6/30 (20.0%)	Undefined	Breast cancer (Antoniou et al. 2003; Easton et al. 1993; Wooster et al. 1995), ovarian cancer (Antoniou et al. 2003), Fanconi anemia (Howlett et al. 2002)
Gaildrat et al. (2012)	*BRCA2* exon 7 (115 bp)	Variants of unknown significance from families undergoing genetic counselling	6/8 (75.0%)	6/8 (75.0%)	Undefined	Breast cancer (Antoniou et al. 2003; Wooster et al. 1995), ovarian cancer (Antoniou et al. 2003), Fanconi anemia (Howlett et al. 2002)
Di Giacomo et al. (2013)	*BRCA2* exon 7 (115 bp)	Variants reported in breast and ovarian cancer patients	7/23 (~30.4%) (includes small indels)	8/26 (~30.8%) (includes small indels)	Undefined	See above
Kergourlay et al. (2014)	*DYSF* (various exons)	Missense mutations reported as disease-causing	5/24 (~20.8%)	5/25 (20.0%)	Undefined	Muscular dystrophy (Bashir et al. 1998; Liu et al. 1998)
*Mueller* et al. (2015)	SMN1 *exon 7 (54* *bp)*	*All possible combinations of synonymous mutations within a sliding 2*-*codon window*	*Not reported*	*32/138 (*~*23.2%)(includes both single and multiple mutations)*	*Bonferroni*-*corrected p value* <*0.05 in a Fisher’s Exact Test comparing the ratio of reads in the input DNA plasmid library to that in the output sample for the wild*-*type sequence vs for the mutant. In addition, PSI could be no more than 70% of wild*-*type levels*	*Spinal muscular atrophy (Lefebvre et al.* 1995 *)*
Soukarieh et al. (2016)	*MLH1* exon 10 (94 bp)	All reported single-base substitutions (most from cancer patients)	13/18 (~72.2%)	17/22 (~77.3%)	PSI more than a single standard deviation removed from that observed in wild-type (standard deviation from three replicates)	See above
*Julien et al.* (2016)	FAS *exon 6 (63* *bp)*	*All possible single and almost all possible double mutations*	*58/63 (*~*92.1%)*	*115/189 (*~*60.8%)*	*p* < *0.05 in Welch’s unequal variances t test comparing wild*-*type to mutant (3 replicates for either)*	*autoimmune conditions (Cheng et al.* 1994 *; Fisher et al.* 1995 *)*
Tajnik et al. (2016)	*FIX (F9)* exon 5 (129 bp)	Haemophilia B associated single-base substitutions, selected either because their disease-causing mechanism was unclear or because they were located in a region thought to contain splice enhancer elements	6/9 (~66.7%)	9/17 (~52.9%)	Undefined	Haemophilia B (Bolton-Maggs and Pasi 2003)

The column entitled *proportion of splice-disrupting variants* reports the fraction of tested variants that were classed as splice-altering. The column *proportion of splice-associated sites* contains the fraction of tested sites in the exon where any splice-altering variants were detected. Unless otherwise noted, only single-base substitutions are considered. The column *definition of splice alteration* details the criteria used in the study for classifying a variant as splice-altering. Only exonic variants are considered

Italicized rows correspond to studies classed here as belonging to the second subtype (studies that chose the variants to test in an unbiased manner)

**Fig. 1:**
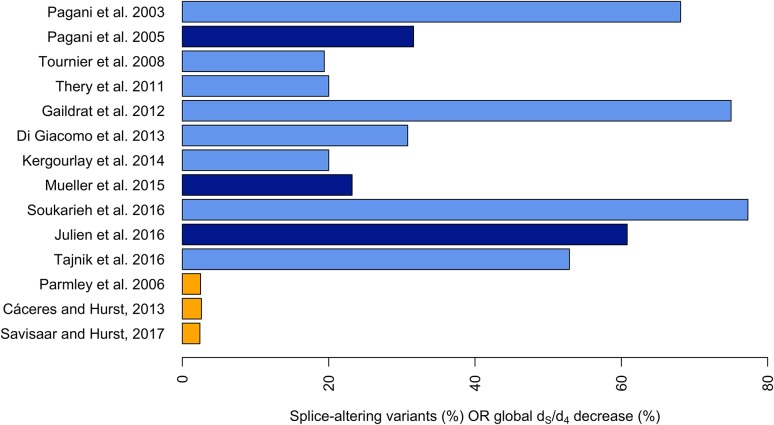
Percentage of splice-altering variants among variants tested (*blue bars*) or over-all percentage decrease in *d*_*S*_ (synonymous rate of evolution)/*d*_4_ (fourfold degenerate rate of evolution) attributed to the need to preserve splice control elements (*orange bars*). The *light blue bars* correspond to subtype 1 (at least some variants chosen because of disease association) and the *dark blue bars* to subtype 2 (largely unbiased selection of variants). There is a large discrepancy between *blue* (experimental) and *orange* (computational) *bars*. Note, however, that the figures are directly comparable only if one assumes that the selection detected in the computational studies is strong enough to preclude all substitutions at selected sites (see “It is uncertain how to infer the density of selected sites from the decrease in *d*_*S*_”). Note also that the estimate from Savisaar and Hurst (2017) reflected selection on non-splice related RNA-binding protein target motifs as well

Such minigene assays have revealed an unexpectedly dense web of splice regulatory information within exons (Fig. 1). All of the studies found at least 19.4% of the assayed exonic mutations to disrupt splice patterns, with a median of ~31.6%. Notably, the only study to assay all possible single-base substitutions within an exon (Julien et al. 2016) reported ~60.8% of the variants to be significantly splice-altering, whilst at least one of the three possible base changes was splice-disrupting at ~92.1% of sites. This led the authors to conclude that “splicing regulatory sequences are distributed across nearly every nucleotide in the exon” (Julien et al. 2016, page 2). On the whole, the splice assays suggest exonic splice regulatory information to be very common and therefore potentially a major driver of exon evolution.

On closer inspection, the splicing assay based studies can be seen to fall into two distinct subtypes, which are implicitly designed to answer different questions. Only one of them is directly relevant to the topic of this manuscript. In the first subtype, most or all of the variants assayed are chosen because they have been observed in disease families (Di Giacomo et al. 2013; Gaildrat et al. 2012; Kergourlay et al. 2014; Pagani et al. 2003; Soukarieh et al. 2016; Tajnik et al. 2016; Thery et al. 2011; Tournier et al. 2008). Enrichment for variants with phenotypic effects is therefore expected. Because of this, these studies do not constitute an unbiased examination of the density of splice regulatory information in the exon. Their results are more relevant to the problem of determining the fraction of exonic disease-causing mutations that owe their effects to splice disruption (although indirectly so: only a subset of the mutations reported in disease families are expected to be pathogenic, especially as several of the studies explicitly consider variants of unknown significance). The values returned for the percentage of splice-disrupting exonic variants range from ~19.4% (Tournier et al. 2008) to ~77.3% (Soukarieh et al. 2016). These figures are consistent with the results from a diverse set of computational and theoretical works that have sought to establish the fraction of splice-altering variants among pathogenic SNPs, and have obtained estimates ranging from about one-fifth to nearly a half (Lim et al. 2011; Sterne-Weiler et al. 2011; Wu and Hurst 2016) (although see Lopez-Bigas et al. (2005), that produce a higher value of approximately 62%).

In the second subtype of studies, the variants are chosen in a largely unbiased manner. In the most extensive experiment of this kind that has been performed so far, the authors generated all possible single base mutations and the majority of possible double mutations (Julien et al. 2016). The two other studies in this category only considered synonymous sites. Mueller et al. (2015) tested all possible combinations of synonymous mutations within a sliding two-codon window moved along the exon, whilst Pagani et al. (2005) assayed a subset of the potential variants within a 40 base pair (bp) stretch of the exon. The fraction of splice-disrupting variants detected ranges from ~23.2% in Mueller et al. (2015) to ~60.8% in Julien et al. (2016). It is only experiments of this second subtype that can directly inform us on the prevalence of exonic splice regulatory information. We have nevertheless discussed both subtypes here, as they superficially appear very similar and it is important to highlight the distinction.

### *k*-mer density and conservation

The second approach to quantifying the importance of exonic splice regulatory information is genome-wide and computational. It is based on the assumption that regulatory signals that overlap CDS should cause a drop in the local rate of synonymous evolution (*d*_*S*_) because variants that disrupt the regulatory motif would be selected against, decreasing the probability of substitutions. Note that evolution is expected to slow down also at non-synonymous sites that overlap splice regulatory elements. However, because it is difficult to disentangle selection on protein primary structure from selection on non-coding information, it is simpler to work with synonymous sites, where variation does not affect the amino acid sequence encoded for.

One proceeds by defining a set of *k*-mers as potentially splice regulatory and then comparing *d*_*S*_ at sites overlapping these motifs to *d*_*S*_ at control sites presumed not to be important for splicing. This provides an estimate for the decrease in evolutionary rate within splice regulatory motifs. It is then possible to infer the over-all impact of the need to preserve splice regulatory information on human *d*_*S*_. To do this, one multiplies the per cent decrease in *d*_*S*_ in motifs vs controls by the fraction of sequence that overlaps the motifs. Importantly, the control sites have to be roughly nucleotide-matched to the sites that overlap splice motifs, or another strategy (such as removal of fast-evolving *CG*/*GC* dinucleotides) needs to be implemented to control for nucleotide composition biases. This is because splice regulatory motifs, notably ESEs, frequently have a highly skewed base content (Cáceres and Hurst 2013). Therefore, if one does not control for such biases and observes motif sites to be slower-evolving than non-motif sites, then one cannot know whether this is due to purifying selection on the motifs or rather to differences in mutation rate between motifs and non-motifs related to differences in nucleotide composition. For instance, motif sites may appear to be slower-evolving if hypermutagenic *CG* dinucleotides are over-represented at non-motif sites.

To our knowledge, there have been three attempts to use such a strategy to quantify the global evolutionary impact of splice regulatory information (Table 2). Two investigated the evolution of different sets of ESEs. Parmley et al. (2006) made use of the RESCUE-ESE hexamers (Fairbrother et al. 2002). These were determined by searching for sequence motifs that were enriched in exons over introns, and near weak splice sites. Some of the motifs were then experimentally validated. Cáceres and Hurst (2013) used several different motif sets. These were obtained by taking the intersection of several previously existing sets of ESEs, including the RESCUE-ESE set, and defining as ESE those *k*-mers that appeared in at least three or four of the pre-existing lists. In addition, we have recently conducted a broader investigation using a large set of motifs experimentally predicted to be recognized by human RBPs (Savisaar and Hurst 2017). This includes both splice factors and other proteins that contact RNA. All of these studies uncovered evidence for evolutionary pressure to preserve the relevant motifs. However, the over-all impact on human *d*_*S*_ was estimated to be a decrease of only 1–4%. This effect is significant and detectable but far weaker than one might expect given the experimental evidence, reviewed above, for the near-omnipresence of splice regulatory information in human exons. In the following section, we will consider four potential explanations for this discrepancy.

**Table 2:** Overview of computational studies on the evolutionary impact of exonic splice regulatory information

References	Motif density	*d*_*S*_/*d*_4_ decrease in motifs	Over-all *d*_*S*_/*d*_4_ decrease	Motifs	Control
Parmley et al. (2006)	~30.42%	~8.19% (including CpG sites)/11.03% (excluding CpG sites) (alignment to mouse)	~2.49% (including CpG sites)/3.36% (excluding CpG sites)	238 RESCUE-ESE ESE hexamers (Fairbrother et al. 2002)	Non-ESE sites
Cáceres and Hurst (2013)	13.1–32.7% (exon ends only)	8.5–17.1% (exon ends only, alignment to mouse)	1.2–4% (extrapolated from exon ends to the full sequence)	Various sets of putative ESEs, formed by taking intersections of pre-existing sets	Either all non-ESE sites near exon ends or sites overlapping with nucleotide-matched control motifs
Savisaar and Hurst (2017)	~57.3%	~4.1% (alignment to macaque)	~2.4%	1483 motifs experimentally determined to be recognized by human RBPs	Sites that overlap dinucleotide-matched control motifs

For Cáceres and Hurst (2013), the figures are presented as a range, as they depend on the set of motifs and the method of control used. Note that some studies considered *d*_*S*_ (rate of evolution at synonymous sites) while others considered *d*_4_ (rate of evolution at fourfold degenerate sites). Parmley et al. (2006) also provided a second estimate for the over-all decrease in *d*_*S*_ (~8%), however, only the lower estimate is reproduced here because of concerns that the reasoning used to derive the higher value may have been circular

*ESE* exonic splice enhancer, *RBP* RNA-binding protein

## Why do the two approaches come to such different conclusions?

### The results from splicing assays might not be representative of the endogenous splicing of most genes

#### The splicing assays are often performed on very short exons

**Fig. 2:**
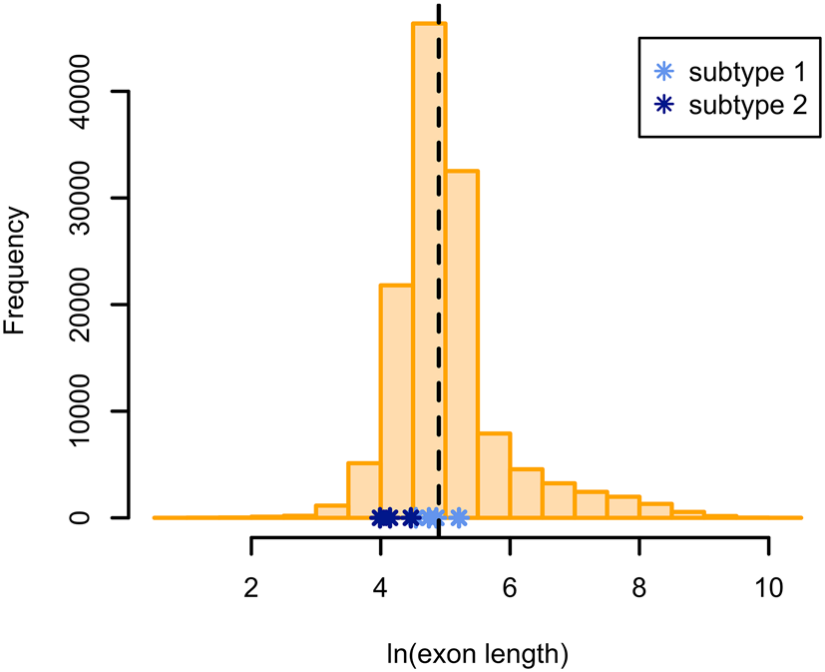
The distribution of exon lengths in the human genome is shown in *orange* (see Online Resource 1 for data). The *dashed line marks* the median of this distribution. The *asterisks mark* the natural logs of the lengths of the exons used in the experimental studies (studies that used more than one exon have been excluded). Note that the majority of these values are below the genomic median, and the three subtype 2 studies (*dark blue*) correspond to particularly low figures

A first explanation is that the splice assays are performed on a specific exon or a few exons, and outside of the normal genomic context. It might therefore be inappropriate to generalize their results to the ‘average’ endogenous gene. Crucially, the exons used in the minigene studies are frequently shorter than average for a human exon (Table 1; our dataset of 10,877 multi-exon protein-coding genes has a median exon length of 134 bp; this dataset was compiled identically to the multi-exon set used in Savisaar and Hurst (2016), except that Ensembl release 85 annotations were used; see Fig. 2 and Online Resource 1). ESEs, and potentially other types of splice regulatory elements, are disproportionately found at the ends of exons (Cáceres and Hurst 2013; Fairbrother et al. 2004; Parmley and Hurst 2007; Woolfe et al. 2010; Wu et al. 2005). The shorter the exon, the larger the proportion of sequence that is close to the splice junction, perhaps leading to a higher density of splice information than would have been observed in a larger exon. The computational studies, on the other hand, would have included the full range of exon sizes, including potentially very large exons (Cáceres and Hurst (2013), who only considered exon ends and then extrapolated to full exons under the assumption of no splice constraint in exon cores, are an exception). The presence of such very large exons, where the majority of the sequence is far from an exon–exon junction, likely decreased the estimated density of splice regulatory elements, widening the gap with experimental estimates.

To what extent does this factor explain the discrepancy? We will define exon end as the 70 bp closest to the exon–exon junction [although ESEs can sometimes function from an even greater distance (Graveley et al. 1998)]. An internal exon has two ends, whilst a terminal exon only has one. Under this definition, all three of the exons used in the experimental studies of subtype 2 were ‘all end’, in that they were all shorter than 141 bp [the exon sizes were 87 bp in Pagani et al. (2005), 54 bp in Mueller et al. (2015) and 63 bp in Julien et al. (2016)]. Therefore, their results pertain to splice regulatory element density specifically at exon ends rather than in exons generally. We will assume that functional splice regulatory information only occurs at exon ends, whilst exon cores are free of splice-related constraint. To generalize from the results of the experimental studies to all coding sequence, we can multiply the fraction of splice-altering variants by the proportion of nucleotides in all ORFs combined that are found within 70 bp of an exon–exon junction. For our gene set, this latter proportion is ~0.348. Therefore, if we take the results of the experimental studies to be representative of most exons, but only for exon ends, then Pagani et al. (2005), Mueller et al. (2015) and Julien et al. (2016) would predict the proportion of splice-altering variants in CDS over-all to be ~11.0, ~8.1 and 21.2%, respectively. This does not fully close the gap with computational estimates, but narrows it considerably. Note, however, that the estimates from Cáceres and Hurst (2013) might also have to be reduced, as they were derived under the assumption that 80% of nucleotides were at exon ends. If instead we use a figure of 34.8%, as for the experimental results, then the 1.2–4% range for the over-all decrease in the rate of evolution at fourfold degenerate sites becomes 0.5–1.7% instead.

Crucially, the figures we have just provided rely on the assumption that exon cores are free of splice-related constraint. This is almost certainly false: a quarter of the splice-altering genomic variants studied by Woolfe et al. (2010) were located in the central parts of exons (although it is unclear how much of this signal was contributed by short exons that would have been ‘all end’ by our definition). In our work, we have found ESEs to be under purifying selection also in the cores of long exons (Online Resource 2). The extent to which correcting for exon sizes closes the gap between the computational and the experimental estimates therefore depends on the difference in functional splice element density between exon cores and exon ends.

#### The splicing assays investigate exons from disease-relevant genes

Another reason why the exons used in the minigene assays may be unrepresentative is that in all eleven studies, they were derived from genes with known disease relevance (Table 1). The functional pressures acting on a disease gene might be different from those that concern other genes and this might lead to somewhat different mechanisms of splice regulation. Importantly, in some cases, changes in the PSI of the exon under study are specifically known to be disease-causing (e.g. Julien et al. 2016; Pagani et al. 2005). The relevance of this problem is hard to pin down until exons that have been chosen in a less biased way have been tested and there is a better understanding of the variation between exons from different genes in terms of their splice regulatory content. Note that the case of Pagani et al. (2005) is particularly interesting. Homozygous mutations in the *CFTR* gene cause cystic fibrosis but heterozygotes seem to be somewhat protected from certain other diseases, such as cholera (Gabriel et al. 1994) and typhoid fever (Pier et al. 1998). It is unclear whether and how such heterozygote advantage may affect the susceptibility of the exon to splice pattern alteration.

#### The focal exon is often flanked by unusually short introns in the minigene

A further issue is that in the minigene studies, the exon has been removed from its natural gene anatomic context. Most notably, ten out of the eleven studies used constructs where the focal exon was flanked by artificial introns—an understandable decision given that the use of the endogenous introns would often be technically challenging or impossible because of their large size. In most cases, hybrid introns were constructed, with the exon proximal 100-200 bp originating from the endogenous intron and the rest from the vector (Di Giacomo et al. 2013; Gaildrat et al. 2012; Kergourlay et al. 2014; Pagani et al. 2003, 2005; Soukarieh et al. 2016; Tajnik et al. 2016; Thery et al. 2011; Tournier et al. 2008). The two exceptions were Mueller et al. (2015), who used shortened versions of the wild-type introns, and Julien et al. (2016), whose study was the only one to use the full introns [presumably because they were quite short to begin with—152 and 1183 bp, according to Ensembl release 85 annotations (Yates et al. 2016)]. The end result of these manipulations is often that the introns used are substantially shorter than those present endogenously. For instance, in Mueller et al. (2015), the upstream intron is reduced from over 6 kb in length to merely 217 base pairs (Singh et al. 2004). In other cases, the change is less drastic but not necessarily trivial: in the construct used for most of the assays in Soukarieh et al. (2016), the upstream and downstream introns span 516 and 2229 bp, respectively (A. Martins, personal communication), whereas the corresponding endogenous lengths are 2961 and 2710 bp (Ensembl release 85 annotations).

These details matter because there is evidence that splicing efficiency or mechanisms of splice control may differ depending on intron size, although the specifics remain unclear (Cáceres and Hurst 2013; Dewey et al. 2006; Fox-Walsh et al. 2005; Hollander et al. 2016; Klinz and Gallwitz 1985; Osella and Caselle 2009; Savisaar and Hurst 2016; Schüler et al. 2014; Sterner et al. 1996; Warnecke et al. 2008b; Wu and Hurst 2015). Human introns tend to be large: our dataset of 10,877 human multi-exon protein-coding genes presents a median intron length of ~1567 bp, with a maximum of 778,855 bp (Online Resource 3). Depending on the study, the introns used in the minigene construct may or may not be unusually small for a human intron. This might have implications as to the generalizability of the results: it is possible that conclusions drawn from an assay that uses 500 bp long flanking introns may not be valid for a more typical human exon flanked by substantially longer introns. This is especially true as both us (Savisaar and Hurst 2016) and Dewey et al. (2006) have detected the presence of a threshold of about 1.5 kb, above which intron size patterns of exonic splice enhancer usage abruptly change. Problematically, none of the nine publications explicitly state the total sizes of the introns in the plasmids used.

Despite this potential issue, it should be emphasized that minigene assays have fared well when their results have been compared to splice isoform ratios in patient RNA. For example, several studies (Bonnet et al. 2008; Thery et al. 2011; Tournier et al. 2008) analysed altogether nearly 90 variants using both a minigene splicing assay and examination of patient RNA, and found good concordance with regards to splice patterns (although note that many of the variants in Bonnet et al. (2008) and Thery et al. (2011) were intronic). Several of the studies considered here (Gaildrat et al. 2012; Kergourlay et al. 2014; Pagani et al. 2003; Soukarieh et al. 2016) included similar comparisons on a smaller scale. They too reported in vivo and reporter data to be largely consistent. In addition, it is routine in splice assay based studies to verify that the reporter recapitulates endogenous exon inclusion levels before introducing mutations into the sequence. Such findings somewhat alleviate the concern that problems such as intron size differences between the minigene and the endogenous gene could be majorly biasing the results obtained from these experiments. However, given that the relationship between splice regulatory mechanisms and gene anatomy is still not well understood, the explanation cannot be discarded and could potentially explain some of the discrepancy between experimental and computational analyses. Most importantly, studies that make use of splice assays should explicitly report both the sizes and the sequence of the different regions of the minigene, along with the dimensions of the corresponding regions in the endogenous gene.

### The splicing assays do not directly test for functional relevance

A second possible explanation is that the splicing assays ultimately test for phenotypic effects and not for functionality (in the sense of visibility to selection; see “A further manifestation of the problematic nature of the term functional?” for further discussion). They can tell us whether a given variant leads to a change in splice form ratios but not whether this change matters to the organism. This point is especially important given that the species considered—human—has a low effective population size (*N*_*e*_) (Tenesa et al. 2007) and so natural selection is expected to be inefficient (Charlesworth 2009). Moreover, for many proteins, the relationship between gene activity and fitness appears to be one of diminishing returns (Jiang et al. 2013; Kacser and Burns 1981; Keren et al. 2016) (note that Kacser and Burns (1981) considered the flux of a pathway rather than fitness directly). In other words, once a certain threshold level of gene expression has been reached, further increases only have a minute effect on fitness. A corollary of this relationship is that levels of functional protein can often be greatly reduced without causing a significant drop in fitness, potentially explaining why most mutations are recessive (Kacser and Burns 1981; Wright 1934). It is therefore possible that for many exons, only the most drastic changes in PSI are selected against while most variation is neutral (or effectively neutral). In addition, mis-splicing events that lead to the introduction of a premature stop codon would at least some of the time likely be caught by nonsense-mediated mRNA decay (NMD) (Brogna et al. 2016; Schweingruber et al. 2013). This would decrease the likelihood of any dominant negative effects due to the presence of truncated protein.

Under this scenario, the gap between experimental and conservation-based estimates would correspond to those mutations that alter splice form ratios but not to an extent that would be visible to natural selection (the function-activity gap). The fraction of mutations that fall into this category depends in part on the stringency of the threshold used for calling a variant as splice-altering in the minigene studies (Table 1). We will now consider each of the three studies of the second subtype (studies where the variants were chosen in an unbiased manner) in turn to determine to what extent the function-activity gap may have inflated the estimates they produced.

Mueller et al. (2015) was the only one of the three publications to explicitly tie the significance threshold to clinical data. The authors set two conditions for an alteration to be defined as a significant splice defect. Firstly, they required the input/output ratio of the reads mapping to the mutant exon to be significantly different from the corresponding ratio obtained in the wild-type. Secondly, and more importantly in the current context, the exon inclusion levels of significantly splice-altering variants could be no more than 70% of those observed for the wild-type sequence. This threshold was set based on the splice defect reported in spinal muscular atrophy patients. It is therefore likely that splice disruptions having an effect this great or greater would be functionally relevant.

Pagani et al. (2005) did not define a formal threshold for what counts as a significant effect on splicing. However, the variants that were categorized as splice-altering reduced exon inclusion from the 80% observed in the wild-type to between 5 and 40%. These are substantial effects, with the residual proportions of full-length transcript similar to those observed in cystic fibrosis and congenital bilateral absence of *vas deferens* (CBAVD) patients (Rave-Harel et al. 1997). A function-activity gap of some extent cannot be ruled out, as exon 12 inclusion levels have been found to vary drastically also among healthy individuals (Slomski et al. 1992). However, the large effect sizes make it unlikely that it could have majorly inflated the final estimate for the proportion of functional variants.

Such inflation is, however, more likely for the dramatic results reported in Julien et al. (2016). In this publication, variants were defined as splice-altering if the PSI significantly differed from wild-type in a Welch’s unequal variances *t* test using distributions obtained over three replicates. From Fig. 2a in Julien et al. (2016), it appears that some of the mutations reported as splice-altering changed the PSI by no more than 5–10%. These effects may be statistically significant but their fitness relevance is uncertain. Note that Mueller et al. (2015) and Pagani et al. (2005), whose results are more likely to be relevant to fitness disruption, provide relatively low estimates for the proportion of variants that alter splicing (23.2–31.6%). These contrast strikingly with the estimate from Julien et al. (2016) (~60.8%), who more liberally defined “splice-altering”.

We emphasize that the argument raised in this section is not a caveat or a criticism—like all experiments, the minigene splicing assays are appropriate for answering some types of questions and not others. However, it is important to be aware of this issue because it limits the extent to which the data from such studies can be used to draw inferences about evolution.

### The conservation-based analyses do not capture all of the evolutionary constraint on splice regulatory information in the exon

#### The conservation-based analyses consider only some of the relevant regulatory signals

The third potential explanation is that the evolutionary analyses are probably inherently biased towards underestimating the total evolutionary constraint. This is so, firstly, because they require a pre-defined list of splicing-relevant motifs. This list will probably never capture all of the splicing information in the exon and some of the relevant sites will therefore not be taken into consideration. Secondly, there are also more technical reasons why some studies of this type might be prone to underestimating the level of constraint, notably when motif density is high. We will consider the first of these issues here and the second in “Certain types of evolutionary analyses are expected to under-estimate the level of constraint when motif density is high”.

A major caveat of the evolutionary studies is that the results will obviously depend on the set of motifs taken to be relevant. Cáceres and Hurst (2013) solely considered putative splice enhancers. This sets their work apart from the minigene analyses, which usually consider both increases and decreases in the extent of exon inclusion, meaning that they are expected to be sensitive to both positive and negative splice regulatory elements. It is possible that including presumed splice repressor motifs in the analysis performed by Cáceres and Hurst (2013) would have led to higher estimates for the evolutionary impact of the motifs. However, the effects reported for splice enhancers are so weak that it seems unlikely that this change alone would have qualitatively altered the conclusions. Indeed, Parmley et al. (2006), although also primarily focused on ESEs, performed a supplementary analysis where they considered splice suppressors as well and reported little additional constraint.

More generally speaking, however, even if one were to consider both splice enhancers and splice repressors, it is likely that the list could never include all of the sequence motifs important for exonic splice regulation. Indeed, as the binding preferences of RBPs usually form a gradient (Jankowsky and Harris 2015), it is likely logically impossible to ever define such an exhaustive set. A further problem relates to the degeneracy of many splice motifs. For instance, a given position in an ESE might accept both an *A* and a *G*. If this position overlaps with a two-fold degenerate site on the pre-mRNA, then the result may be a synonymous site that is functional in splicing but whose functionality does not affect *d*_*S*_ (although it would cause a drop in the non-synonymous rate of evolution, if changes to *T* or *C* disrupted the ESE). Such sites would not contribute to the signal of constraint detected in the evolutionary studies.

Moreover, not all of the splice regulatory information in the exon can be represented as *k*-mers. For example, there is evidence that the GC content differential between exons and introns can affect exon inclusion levels (Amit et al. 2012). Another potential source of splice-related constraint is selection pressure on pre-mRNA secondary structure. For instance, research suggests that for certain RBPs, selection to ensure binding could extend beyond the actual target site, and act on the surrounding sequence so as to maintain the site single-stranded and thus accessible (Hiller et al. 2007; Jin et al. 2014; Liu et al. 2010; McManus and Graveley 2011). Other RBPs, conversely, preferentially bind structured elements, such as hairpins (e.g. Aviv et al. 2006). Pre-mRNA secondary structure may play a role in splicing also by concealing the splice sites of alternatively spliced exons (Shepard and Hertel 2008; Zhang et al. 2011). Importantly, Meyer and Miklos (2005) showed that the mutations that induced greater splice defects in the Pagani et al. (2005) minigene study led to somewhat greater alterations in pre-mRNA secondary structure than mutations that affected splicing less. Some of the instances of exon skipping observed in Pagani et al. (2005) may therefore have been due to changes in RNA secondary structure. The relevant sites would not have been captured in the evolutionary analyses. Yet other mechanisms of control relate to the fact that human splicing appears to be at least partly co-transcriptional (de Almeida and Carmo-Fonseca 2014; Gomez Acuna et al. 2013; Hollander et al. 2016; Nojima et al. 2015). The elongation rate of RNA polymerase II (Fong et al. 2014; Saldi et al. 2016), nucleosome positioning (Fong et al. 2014; Saldi et al. 2016), histone modifications (Andersson et al. 2009; Kolasinska-Zwierz et al. 2009) and CpG methylation (Chodavarapu et al. 2010; Laurent et al. 2010; Lev Maor et al. 2015; Wan et al. 2013; Yearim et al. 2015) might all be involved in determining splicing outcomes.

For the reasons stated above, it is highly probable that there are splice regulatory sites in exons that are not considered in the evolutionary analyses discussed here. This may partially explain the discrepancy with the results from splice assay based studies (although, given that the splice assays are performed on a plasmid rather than a chromosome, they might also be insensitive to some of the types of information considered above). On the other hand, the proteins that bind ESEs also have roles in processes other than splicing (Änkö 2014; Howard and Sanford 2015; Huang et al. 2003; Ji et al. 2013; Li and Manley 2005; Lin et al. 2008; Maslon et al. 2014; Michlewski et al. 2008; Sanford et al. 2004; Sapra et al. 2009; Swartz et al. 2007; Taniguchi et al. 2007; Wu et al. 2010). It is therefore likely that part of the conservation signal observed at ESE sites is splicing unrelated, a conclusion supported by the fact that ESEs are enriched and conserved also in intronless genes (Pozzoli et al. 2004; Savisaar and Hurst 2016). This might, conversely, lead the ESE-based methods to over-estimate- the frequency of sites that function in splicing. It is unclear to what extent this opposing tendency might mitigate any under-estimation due to relevant sites being ignored. Note that these methods also have a high false positive rate in the sense that many motif hits may not have any non-coding functions at all (whether in splicing or in other processes). This might dilute out the conservation signal from true positives. However, in the context of estimating over-all constraint, this is less troubling than false negatives (see “Summary and future directions” for further discussion).

#### Certain types of evolutionary analyses are expected to under-estimate the level of constraint when motif density is high

In addition to concerns regarding the repertoire of sites considered to be relevant, it is possible that some ways of defining the control sites may inherently lead to under-estimation of the constraint. This caveat is particularly relevant to the method used in Savisaar and Hurst (2017). In this publication, we generated 1000 sets of simulant motifs of the same size and roughly the same dinucleotide composition as the true motifs that were being investigated. We then calculated *d*_*S*_ at sites overlapping the simulant motifs and used the mean of this distribution as the null expectation, relative to which to call excess conservation. The advantage of this method is that it controls for motif number, length and (di)nucleotide composition. However, its conservativeness might become a problem when motif density is high. For instance, we can imagine that the results reported for *FAS* exon 6 in Julien et al. (2016) were indeed representative of the endogenous splice control of the average human exon. The authors reported that ~92.1% of all sites harboured at least one splice-altering variant. Reformulated in the framework of the evolutionary analyses, this would mean that the density of splice regulatory elements within exons was ~92.1%.

In the unlikely case that all of this splice information could be detected by searching for a set of *k*-mers, the set would necessarily have to be very large if it is to overlap with ~92.1% of the sequence (although the lower *k*, the smaller the set can be). For example, the set of putative RBP target motifs used in Savisaar and Hurst (2017) is composed of 1483 motifs and only has a median density of ~57.3% in human CDSs. The simulant sets have the same size as the true set and would therefore also have to be very large. Any set composed of thousands of hexamers (or other *k*-mers of a similar *k*) will have a high density in any exon, simply because the number of possible hexamers is limited. This is even more so the case if the simulants are dinucleotide-matched to functional exonic motifs. The upshot is that if the vast majority of the sequence overlaps with true motifs, then the vast majority will also overlap with the simulants. Many sites will thus be shared between the motifs and the simulants. The decrease in *d*_*S*_ in the motifs might be highly significant (based on the empirical distribution from the simulants) but the effect size is necessarily going to be small because of this prevalence of shared sites. Therefore, if the density of functional sites is high, this methodology will under-estimate the true evolutionary impact.

We conclude that the results produced in Savisaar and Hurst (2017) potentially under-estimate the true extent of the evolutionary impact of the need to preserve RBP target motifs. However, the two other evolutionary studies (Cáceres and Hurst 2013; Parmley et al. 2006) used a different approach for calculating baseline conservation levels. In these cases, the authors simply compared motif to non-motif sites (removing potentially hypermutagenic *CG*/*GC* dinucleotides in Parmley et al. (2006)). Although such an approach is more prone to nucleotide composition biases, it should not exhibit the issue considered in this section. Despite this, these studies also reported the evolutionary effect of splice motif preservation to be weak (Table 2). Therefore, although some techniques for estimating excess conservation are likely inappropriate in situations where the motif density is very high, this factor is unlikely to explain the discrepancy.

### It is uncertain how to infer the density of selected sites from the decrease in *d*_*S*_

**Fig. 3:**
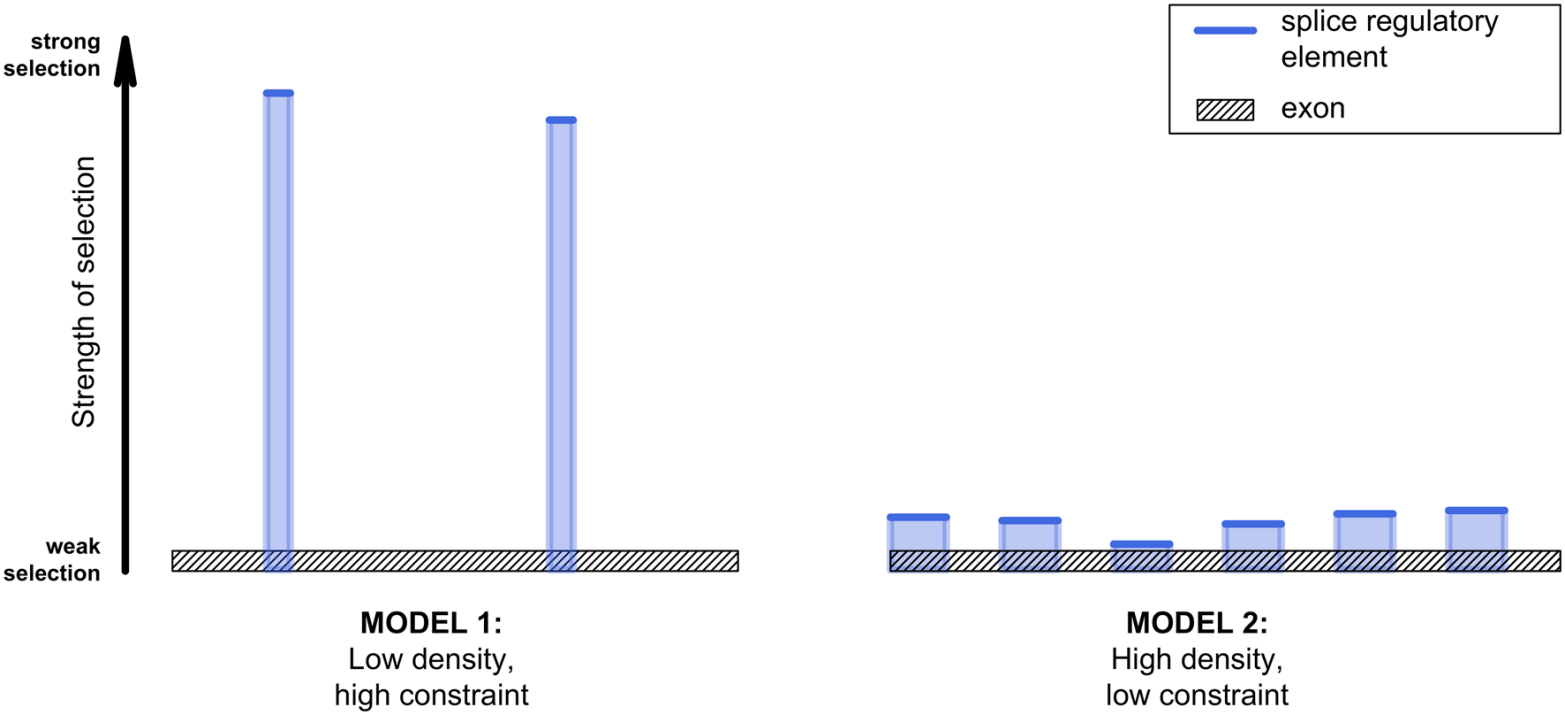
Two models for the distribution of functional splice regulatory information along the exon. Under the first model, functional splice regulatory elements are rare but under strong purifying selection. Under the second model, functional splice regulatory elements are frequent but only weakly constrained. Intermediate scenarios are also possible

The fourth explanation is that the evolutionary analyses do not inform us on how the purifying selection that they detect is distributed across *k*-mer hits. This makes it difficult to compare their results with those from experimental assays. One possible interpretation of the estimated 1–4% decrease in *d*_*S*_ is that only 1–4% of synonymous sites overlap with functional splice regulatory elements (Model 1 in Fig. 3). The purifying selection acting at these sites would then have to be very strong, with the fixation probability of incoming mutations at 0. All other motif hits would be false positives. If this was the case, then the discrepancy with experimental estimates would be astounding.

At the other extreme, it could be that all of the motif hits function in splicing and that there are therefore no false positives (Model 2 in Fig. 3). Under this scenario, the purifying selection acting at these sites would be too weak to bring fixation probability to 0, but strong enough to somewhat decrease it, leading to the signal of purifying selection. This would put the density of functional splice regulatory elements at somewhere between 13 and 57%, depending on the study considered (although note that the higher end of the range is from work that also considered RBPs that are not thought to be splice factors). These figures are compatible with the lower experimental estimates (Table 1). The truth may also lie midway. The motif hits might be made up of three classes of sites: those not undergoing splice-associated purifying selection (false positives), those under strong purifying selection (no substitutions allowed) and those under weak purifying selection (substitutions possible but less likely than for controls).

Ideally, one would distinguish between these models by estimating evolutionary rates separately for each motif hit. However, sites that differ between closely related species are too rare for such an analysis to be feasible. Polymorphism-based approaches suffer from the same limitation, as SNPs are also rare. For both methodologies, many sites therefore have to be pooled to have enough information for reliable estimation. Various approaches have been developed to provide more fine-scale conservation information, sometimes up to single base resolution. These mostly use multiple sequence alignments between a large number of species (e.g. Lin et al. 2011; Pollard et al. 2010; Siepel et al. 2005). However, such analyses are expected to only be sensitive to selection acting over long evolutionary time scales (Ponting and Hardison 2011). This is a serious drawback for studies of exonic splice information, as splice patterns can rapidly change (Alekseyenko et al. 2007; Nurtdinov 2003; Pan et al. 2005).

However, even if it is not possible to obtain conservation estimates for individual putative splice regulatory elements, it may be feasible to estimate the over-all distribution of fitness effects (DFE) (Eyre-Walker and Keightley 2007) at these sites. The DFE is the distribution of selective coefficients among new mutations. Older approaches estimated this from divergence information alone (Nielsen and Yang 2003), or by combining divergence data with a summary estimate of polymorphism levels (Loewe et al. 2006; Piganeau and Eyre-Walker 2003; Sawyer et al. 2003). More recent studies tend to make fuller use of the observed allele frequencies (Boyko et al. 2008; Eyre-Walker et al. 2006; Gronau et al. 2013; Keightley and Eyre-Walker 2007; Keightley and Halligan 2011; Kousathanas and Keightley 2013; Lawrie et al. 2013; Schneider et al. 2011; Wilson et al. 2011). Briefly, these studies typically define a focal class of sites where at least some mutations are thought to affect fitness and a second class of sites presumed to be evolving neutrally. The most likely parameters for the DFE at the focal sites are then estimated from the observed divergence and/or polymorphism data, while the neutral class serves as control and can be used to determine parameters related to demography and the mutation rate.

Although DFEs have mostly been obtained for amino-acid changing mutations, synonymous sites have also been analysed (Keightley and Halligan 2011; Lawrie et al. 2013; Racimo and Schraiber 2014). In addition, Gronau et al. (2013) devised INSIGHT, a method designed specifically for analysis of short interspersed non-coding regions. It has been used so far for the analysis of transcription factor binding sites (Arbiza et al. 2013), microRNAs (Gronau et al. 2013), small nucleolar RNAs (snoRNAs) (Gronau et al. 2013) and long intergenic non-coding RNAs (lincRNAs) (Gronau et al. 2013). Extending this work to fourfold degenerate sites that overlap putative splice regulatory elements would be a natural next step. Importantly, the definition of the baseline class of sites would have to be different from that used in previous studies. Whereas typically, the control sites are derived from genomic regions presumed to be evolving neutrally, the analysis envisaged here would entail also using a secondary control corresponding to coding sites not thought to be involved in splice regulation. This is necessary so as to disentangle selection on splice information from other selective pressures acting at synonymous sites. Note also that many of the problems that are relevant to the evolutionary analyses summarized in Table 2 must also be taken into account when estimating the DFE. This includes notably the definition of relevant sites and the need to control for nucleotide composition biases.

Finally, it should be pointed out that there are important problems related to the distribution of selective coefficients across sites that even knowing the DFE would not automatically solve. Notably, the DFE would not inform us on how evenly functional splice information is distributed among different exons and different genes. This issue is directly relevant to the problem of the representativeness of the exons used in the splice assays (“The results from splicing assays might not be representative of the endogenous splicing of most genes”). A potential clue comes from comparisons of synonymous evolutionary rates in constitutively and alternatively spliced exons. One would expect any differences in levels of conservation between these two classes of exons to reflect splice-associated selection. It is therefore remarkable that synonymous sites in constitutively spliced exons have been found to evolve at about twice the rate of those exhibiting conserved alternative splicing (Parmley et al. 2006; Xing and Lee 2005), with even greater differences if only minor form alternatively spliced exons are considered (Xing and Lee 2005). The studies only analysed cases of conserved alternative splicing, which might make the relevant exons unrepresentative of alternatively spliced exons more generally. However, these findings suggest that there does exist a subset of exons where at least half the synonymous sites overlap splice regulatory elements. This proportion may be even higher if the purifying selection acting on some of these sites is very weak. Importantly, the difference in evolutionary rates between constitutively and (conserved) alternatively spliced exons does not seem to be explained by ESE preservation (Parmley et al. 2006), further underlining the possibility that the computational approaches are missing out on large amounts of relevant information (see “The conservation-based analyses consider only some of the relevant regulatory signals”).

In conclusion, without knowing how the purifying selection observed at presumed splice regulatory *k*-mers distributes across the hits, it is difficult to directly compare the results obtained via *k*-mer searching with those returned from splice assays. However, whatever be the properties of the DFE, the evolutionary studies suggest that the evolutionary impact of motif conservation is either strong and localized, or weak and diffuse. It is hard to imagine how any DFE obtained for these sites would be compatible with selective pressure that is both strong and concerns most of the sequence, and that would thus be a major source of constraint on exon evolution (although note the caveats discussed in “The conservation-based analyses do not capture all of the evolutionary constraint on splice regulatory information in the exon”).

## Concluding remarks

### Summary and future directions

In this review, we have pointed out a remarkable discrepancy between results from evolutionary and experimental studies on the prevalence of functional splice regulatory information within exons. The former have concluded that purifying selection on exonic splice regulatory information is detectable but weak and unlikely to play a major role in directing exon evolution. The latter, on the other hand, have found that mutations all across the exon have great potential to disrupt splice patterns, suggesting that exon evolution may be substantially constrained by the need to ensure correct splicing. Which of these two perspectives is more accurate has major implications for our understanding of how synonymous sites evolve, how (and how often) exonic mutations lead to disease and how synonymous sites should be handled when designing transgenes. We have therefore sought to understand the two methods in detail. We have asked to what extent the discrepancy is real and to what extent it is a result of the two types of analyses asking different questions and measuring different quantities.

We have discussed four potential explanations, all of which are likely to play some role in the discrepancy. Firstly, the results from splice assays may not be representative of typical splice control, as the exons used are often atypically short, derive from disease-associated genes and are analysed outside of their normal gene anatomic context. We emphasize particularly the issue of using short exons, which means a large fraction of the sequence is close to an exon–exon junction, potentially leading to more concentrated splice information than usual. Secondly, the splice assays do not directly measure functional relevance but rather simply changes in splicing patterns, which may or may not be visible to natural selection. Third, conservation-based analyses are liable to under-estimation of the constraint, both because they necessarily only consider a limited set of elements and because in some cases, they can be overly conservative when motif density is high. And finally, the estimates returned from the evolutionary studies are difficult to interpret because we do not know how the detected constraint is distributed across the different elements.

This list of four is not exhaustive and other drivers of the discrepancy could be envisaged. For example, Julien et al. (2016) uncovered widespread epistasis among mutations with regards to their role in splicing. The authors suggested that this could lead to the conservation of splice form ratios all while sequence diverges, and could help “explain why sequence conservation can be a poor indicator of functional importance in exonic regulatory sequences” (Julien et al. 2016). However, this suggestion should be formalized in a more explicit model before its merits can be fully appreciated. Furthermore, Julien et al. (2016) report a very high density of splice regulatory information even if epistasis is not taken into account (the ~92.1% estimate for the fraction of sites relevant to splicing was obtained based on single mutations alone). Therefore, epistasis is unlikely to explain a substantial fraction of the discrepancy observed between experimental and conservation-based estimates for the density of functional splice regulatory elements.

Future work will have to determine the relative importance of the different factors we have discussed. With time, approaches that are even more large-scale than that used in Julien et al. (2016) will likely become possible. This will allow for greater numbers of exons to be assayed. If these exons are chosen independently of their disease relevance or level of conservation, we will as a result obtain a better understanding of the properties of the ‘average’ exon. It will then also be possible to explicitly test the effects of variation in gene architecture on the presence of splice regulatory information. Such analyses will help us determine the extent to which current estimates may be biased by the choice of exons used.

It is also important to improve the evolution-based methods so as to alleviate some of the issues discussed in the present manuscript. Notably, if possible, a normalization method should be used that accurately controls for nucleotide composition without becoming overly conservative at high motif densities (see “Certain types of evolutionary analyses are expected to under-estimate the level of constraint when motif density is high”). It may also be feasible to improve the accuracy of the method and thereby to decrease estimation noise. The *k*-mer searching based methods considered here can only be used to detect *en masse* deviations from null in terms of motif density or conservation. They are not appropriate for pinpointing individual RBP target sites, as primary sequence is only one determinant of where an RBP binds. The mRNA secondary structure (Cook et al. 2015; Li et al. 2010, 2014), and co-operation or competition with other proteins binding in the region (e.g. Pandit et al. 2013; Zarnack et al. 2013) are examples of other prominent factors that also play a role. Motif-based methods do not consider these variables and therefore suffer from a high false positive rate. More sophisticated approaches that are less affected by this issue have been developed. Unlike simple *k*-mer searching, these methods were designed to predict individual RBP binding sites. They go beyond the motif content alone, for instance by taking into account site accessibility (Li et al. 2010; Zhang et al. 2013) or clustering (Akerman et al. 2009; Zhang et al. 2013). Incorporating elements from such approaches may be useful but must be accompanied by a strategy to control for nucleotide composition biases. Moreover, in the present context, false negatives are more detrimental than false positives. The presence of false positives will increase motif density but will also decrease excess conservation, and is therefore expected to have little effect on the estimation of the over-all evolutionary impact. False negatives, however, will decrease density without affecting excess conservation, leading to an underestimation of the global decrease in evolutionary rates. Therefore, decreases in the false positive rate are only desirable if they do not come at the cost of an increased false negative rate.

The various caveats we have discussed are still too severe for us to propose a confident estimate for the true frequency of functional exonic splice information. However, whatever be that true figure, it presumably cannot be higher than the fraction of selected synonymous sites overall. Studies that seek to establish this latter estimate could therefore, in theory, give us an upper bound. In practice, results from different studies are currently too divergent to provide a definitive answer. Certain authors have compared substitution rates at synonymous sites to substitution rates at a presumed neutral control, such as ancestral repeats. They have concluded that 20–25% of synonymous sites are under purifying selection (Eory et al. 2010; Keightley et al. 2011) [though see also Price and Graur (2016), who argue that few if any synonymous sites are under selection]. However, these studies assumed that all mutations were either neutral or sufficiently deleterious to preclude substitutions completely. This means that the figures returned could be under-estimates if certain mutations are only very weakly deleterious. It is therefore more informative to consider studies that have estimated the DFE. Keightley and Halligan (2011) inferred about 30% of mutations at fourfold degenerate sites to be subject to purifying selection [with the product of the effective population size and the selective coefficient (*N*_*e*_ *s*) > = 0.1], and ~11% to be under strong purifying selection (*N*_*e*_ *s* > 10). This is roughly comparable to estimates obtained for Drosophila (Lawrie et al. 2013), although the latter work found no evidence for weakly deleterious mutations. A much higher estimate was produced by Racimo and Schraiber (2014), whose work suggests that about 58.07% of variants at human four-fold degenerate sites are under purifying selection (*N*_*e*_ *s* > = 0.1, assuming *N*_*e*_ = 10,000). However, strong purifying selection would be rare at fourfold-degenerate sites, with 0% of mutations subject to *N*_*e*_ *s* > = 0.5. An important task for the future will be, firstly, to understand why different methods give such different estimates for the fraction of synonymous sites under selection, and secondly, to establish the extent to which the selection that has been detected is splice-related. Determining the distribution of fitness effects at splicing-relevant sites, as discussed in “It is uncertain how to infer the density of selected sites from the decrease in *d*_*S*__*”*_, will be crucial in this regard.

### A further manifestation of the problematic nature of the term functional?

The experimental approach can report on the proportion of sites where mutations lead to splice disruption. A disadvantage of the method that cannot trivially be rectified is its inability to estimate the proportion of sites that are functionally relevant. However, here, as elsewhere in the manuscript, we presume functional to be equivalent to visible to selection. In other words, a sequence element is functional if its disruption decreases fitness sufficiently that natural selection can act upon this decrease. Alternatively, one could consider an element to be functional if its disruption has a phenotypic effect, independently of whether this effect is visible to selection. From this point of view, sites where mutations disrupt splicing would all be functionally relevant by definition.

These two contrasting perspectives echo discussions in the philosophy of biology with regards to what it means for something to have a particular function. A first interpretation, known as the causal role definition of function, considers that a function of a trait is an effect that it has that contributes to a capacity exhibited by a larger system that the trait is part of (Amundson and Lauder 1994; Cummins 1975). For instance, the function of an ESE is to promote exon inclusion if by doing so, it contributes to the splicing machinery’s capacity to splice correctly. The splice assays test which nucleotides contribute to establishing an exon’s percentage of inclusion. These studies therefore inform us on the fraction of nucleotides that have a causal role function in splicing.

An alternative point of view is the so-called selected effects definition of function, which holds that a function of a trait is an effect that it has that has positively contributed to the fitness of previous possessors of that trait, leading to the trait’s persistence in evolution (Garson 2011; Godfrey-Smith 1994; Millikan 1989; Neander 1991). For instance, the function of an ESE is to promote exon inclusion if it has been selected because of its capacity to do so. The evolutionary analyses look for evidence that sequence elements have been selectively maintained over evolution and therefore inform us on selected effects function. The selected effects definition is arguably more widely accepted both in the philosophy of biology and in evolutionary biology. Leaving aside philosophical considerations, an important reason to prefer this definition in the current context is that cellular processes are often error-prone. Many of the events occurring in a cell may be due to processes like leaky transcription, or the spurious binding of proteins to nucleic acids (Pickrell et al. 2010; Struhl 2007). A selected effects definition does not consider these events as functional, even if they are specific, repeatable and relevant to phenotype [see the thought experiment of performing a Random Genome Project in Eddy (2013)]. One way of interpreting the discrepancy between the results from experimental and conservation-based studies is therefore that it is a measure of the extent to which mis-splicing is invisible to selection (although, as discussed in “Why do the two approaches come to such different conclusions?”, several other factors may also contribute to the discrepancy).

This issue of how to define function was hotly debated after the ENCODE Project Consortium (2012) claimed that 80% of the human genome was functional (Doolittle 2013; Eddy 2012, 2013; Germain et al. 2014; Graur et al. 2013, 2015; Hurst 2013; Kellis et al. 2014; Mattick and Dinger 2013; Niu and Jiang 2013; Stamatoyannopoulos 2012). ENCODE considered that residues were functional if they showed particular kinds of biochemical activity or were in the vicinity of sites that did (phenomena such as transcription, protein binding and *CpG* methylation were considered) (ENCODE Project Consortium 2012). This position was heavily criticized (Doolittle 2013; Eddy 2012, 2013; Graur et al. 2013; Hurst 2013; Niu and Jiang 2013), and we note that as it requires evidence for neither causal effect nor selection, it passes the bar neither as a causal role nor as a selected effects definition of function. We hope that in the present manuscript, we have been able to show that careful consideration of what we mean when we claim something to be functional is important above and beyond the debate around ENCODE, as it severely effects how studies are to be designed and the results interpreted.

## Electronic supplementary material

Below is the link to the electronic supplementary material.

Online Resource 1: Transcript identifiers and the lengths of corresponding exons. Used for calculating median exon length (tsv 3810 kb)

Online Resource 2: Brief overview of evolutionary rate calculations in exon cores and flanks (PDF 241 kb)

Online Resource 3: Transcript identifiers and the lengths of corresponding introns. Used for calculating median intron length (tsv 2668 kb)
