# Supplementary material for: Estimating the prevalence of functional exonic splice regulatory information
Source: Hum Genet. 2017 Apr 12;136(9):1059–78. doi: 10.1007/s00439-017-1798-3 (PMC5602102; doi:10.1007/s00439-017-1798-3)
Supplement: Supplementary file 2 — Online Resource 2: Brief overview of evolutionary rate calculations in exon cores and flanks (PDF 241 kb) [file 439_2017_1798_MOESM2_ESM.pdf]

Article: Estimating the prevalence of functional exonic splice regulatory information.
Journal: Human Genetics.
Authors: Rosina Savisaar and Laurence D. Hurst.
Correspondence to: Rosina Savisaar, r.savisaar@bath.ac.uk, The Milner Centre for Evolution, Department of Biology and Biochemistry, University of Bath, Bath, BA2 7AY, United Kingdom

Supplementary Text 1
Selection on exonic splice enhancers in exon cores

We asked whether exonic splice enhancers (ESEs) were under purifying selection also in exon cores (see section 3.1.1. in main text for context). We assembled a dataset of human internal exons that were at least 211 base pairs long (see Savisaar and Hurst (2016), section *Comparing ESE Density in Exon Flanks and Exon Cores* in the *Materials and Methods* for full details on the dataset). We then predicted hits to the INT3 set of ESEs (Cáceres and Hurst 2013) and concatenated the hits from different exons for three exon compartments separately (5' exon flanks, exon cores and 3' exon flanks). We then calculated the synonymous rate of evolution (d_S) in each compartment and normalized it by the distribution of d_S values obtained with 10,000 sets of roughly dinucleotide-matched simulated motifs (see *Materials and Methods* in Savisaar and Hurst (2016) for full details on the simulant motifs and on calculating d_S; $normalized\ d_S = \frac{true\ d_S - mean\ of\ simulated\ d_S}{mean\ of\ simulated\ d_S}$).

We concluded that not only was there evidence for purifying selection on exon cores, this evidence was stronger for cores than for 3' flanks. Several previous publications have provided evidence that ESE function is particularly relevant to exon flanks (see main text for references) and we therefore still maintain that the use of short exons is a likely cause of bias in minigene assays (see section 3.1. in main text). However, the effect is likely to be weaker than suggested by the tentative numbers we have put forth in section 3.1.

	d_S	normalized d_S	empirical *p*-value
5' flanks	~0.054	~−0.131	~0.002
cores	~0.054	~−0.101	~0.014
3' flanks	~0.058	~−0.076	~0.058

Cáceres EF, Hurst LD (2013) The evolution, impact and properties of exonic splice enhancers. Genome biology 14:1–18

Savisaar R, Hurst LD (2016) Purifying Selection on Exonic Splice Enhancers in Intronless Genes. Mol Biol Evol 33:1396-1418
